# PP1, PKA and DARPP‐32 in breast cancer: A retrospective assessment of protein and mRNA expression

**DOI:** 10.1111/jcmm.16447

**Published:** 2021-05-15

**Authors:** Behnaz Saidy, Shreeya Kotecha, Anna Butler, Emad A. Rakha, Ian O. Ellis, Andrew R. Green, Stewart G. Martin, Sarah J. Storr

**Affiliations:** ^1^ Division of Cancer and Stem Cells Nottingham Breast Cancer Research Centre School of Medicine University of Nottingham Biodiscovery Institute Nottingham UK

**Keywords:** DARPP‐32, PKA, PP‐1

## Abstract

Cyclic AMP–dependent protein kinase A (PKA) and protein phosphatase 1 (PP1) are proteins involved in numerous essential signalling pathways that modulate physiological and pathological functions. Both PP1 and PKA can be inhibited by dopamine‐ and cAMP‐regulated phosphoprotein 32 kD (DARPP‐32). Using immunohistochemistry, PKA and PP1 expression was determined in a large primary breast tumour cohort to evaluate associations between clinical outcome and clinicopathological criteria (n > 1100). In addition, mRNA expression of PKA and PP1 subunits was assessed in the METABRIC data set (n = 1980). Low protein expression of PKA was significantly associated with adverse survival of breast cancer patients; interestingly, this relationship was stronger in ER‐positive breast cancer patients. PP1 protein expression was not associated with patient survival. PKA and PP1 subunit mRNA was also assessed; *PPP1CA*, *PRKACG* and *PRKAR1B* were associated with breast cancer–specific survival. In patients with high expression of DARPP‐32, low expression of PP1 was associated with adverse survival when compared to high expression in the same group. PKA expression and PP1 expression are of significant interest in cancer as they are involved in a wide array of cellular processes, and these data indicate PKA and PP1 may play an important role in patient outcome.

## 1 BACKGROUND

Cyclic AMP–dependent protein kinase A (PKA) and protein phosphatase 1 (PP1) are multifunctional proteins involved in numerous essential signalling pathways that modulate physiological and pathological functions which have been implicated in cancer development and progression. Both PKA and PP1 are oligomeric enzymes composed of catalytic and regulatory subunits. PKA is a heterotetramer of two catalytic (encoded by *PRKACA*, *PRKACB*, *PRKACG*) and two regulatory (encoded by *PRKAR1A*, *PRKAR1B*, *PRKAR2A*, *PRKAR2B*) subunits. PKA is involved in a wide array of cellular processes, including metabolism, gene expression and apoptosis. PKA is also expected to play a variety of roles dictated by cellular context due to its influence of multiple cellular signalling pathways. Interestingly, in breast cancer, PKA is capable of phosphorylating serine 305 of oestrogen receptor α (ERα) to induce tamoxifen resistance and alter the transcriptome through redirection of ERα to new transcriptional start sites.^1^, ^2^ Low *PRKAR1A*/high *SRC* mRNA has been shown to define basal‐like and HER2‐positive breast cancers with a worse clinical outcome,^3^ and PKA activity has also been implicated in HER2 resistance in vitro, in particular down‐regulation of the PKA‐RIIα subunit.^4^

Like PKA, PP1 is a multifunctional protein involved in a large array of important cellular pathways. PP1 is an oligomeric enzyme that contains one catalytic subunit (encoded by *PPP1CA*, *PPP1CB* and *PPP1CC*) and at least one regulatory subunit (16 regulatory subunits). Importantly, PP1 plays a role in mitotic regulation,^5^ glycogen metabolism and RNA splicing. In cancer, PP1 has been shown to play a role in the tumour microenvironment ^6^ and in steering cellular migration,^7^ and it has been shown to interact with BRCA1.^8^

Both PP1 and PKA can be inhibited by dopamine‐ and cAMP‐regulated phosphoprotein 32 kD (DARPP‐32), also known as protein phosphatase one regulatory subunit 1B and encoded by *PPP1R1B*. DARPP‐32 was originally described as a signalling protein highly concentrated in regions of the brain enriched in dopaminergic nerve terminals.^9^ DARPP‐32 is able to inhibit both PKA and PP1 dependent upon its phosphorylation status. For instance, DARPP‐32 is phosphorylated on threonine 34 by PKA to allow it to act as a potent inhibitor of PP1.^10^ Cyclin‐dependent kinase (Cdk)‐5 can phosphorylate DARPP‐32 on threonine 75 to allow it to act as a PKA inhibitor.^11^ In a variety of cancers, a truncated DARPP‐32 splice variant (t‐DARPP) is expressed; importantly, this isoform lacks the ability to inhibit PP1, but retains PKA inhibitory activity.^12^ Phosphorylation of t‐DARPP has been shown to mediate PKA activation, which appears to be via association between t‐DARPP and the regulatory R1 subunit.^13^

There is accumulating evidence that differential expression of DARPP‐32 and t‐DARPP is functionally significant in numerous tumour types (reviewed in^14^). Low levels of DARPP‐32 protein expression are associated with shorter cancer‐specific survival of breast and ovarian cancer patients.^15^, ^16^ In trastuzumab‐resistant breast cancer cells, t‐DARPP activated IGF‐1R signalling, which stimulated glycolysis and conferred trastuzumab resistance.^17^ In gastric cancer, DARPP‐32 is frequently overexpressed in the early stages of gastric cancer.^18^ In lung cancer, DARPP‐32 and t‐DARPP promoted tumour cell growth in vivo and in vitro.^19^, ^20^ In pancreatic cancer, loss of HIF1α increased *PPP1R1B* expression, and DARPP‐32 promoted degradation of p53 through phosphorylation of MDM2.^21^

DARPP‐32 is phosphorylated by PKA or Cdk5 to alter its inhibitory activity; Cdk5 is involved in neuronal maturation but is also implicated in cancer and neurodegenerative disorders. In breast cancer, low Cdk5 expression has also been shown to be associated with adverse survival of breast cancer patients.^16^

In breast cancer, we hypothesize that a reduction in DARPP‐32 and an increase in t‐DARPP in breast cancer result in a concomitant alteration of PKA and PP1 signalling. We sought to determine the expression of PKA and PP‐1 mRNA and protein in a large cohort of early‐stage invasive breast cancer patients to understand their relationship between survival and clinicopathological criteria and to determine their relationship between DARPP‐32 and Cdk5 expression.

## 2 METHODS

### 2.1 Patient cohorts

Breast cancer patients were all treated at Nottingham University Hospitals between 1998 and 2006. Patients underwent a wide local excision or mastectomy, decided by disease characteristics or patient choice, followed by radiotherapy if indicated. Nottingham Prognostic Index (NPI), ER and menopausal status determined if patients received systemic adjuvant treatment. Patients with an NPI score less than 3.4 did not receive adjuvant treatment, and patients with an NPI score of 3.4 and above were candidates for CMF combination chemotherapy (cyclophosphamide, methotrexate and 5‐fluorouracil) if they were ER‐negative or pre‐menopausal; and hormonal therapy if they were ER‐positive.

PKA mRNA expression and PP‐1 mRNA expression were determined in the Molecular Taxonomy of Breast Cancer International Consortium (METABRIC) data set (n = 1980).^22^ DNA and RNA were isolated from samples and hybridized to the Affymetrix SNP 6.0 and Illumina HT‐12 v3 platforms for genomic and transcriptional profiling as described by Curtis et al.^22^ Tumours were collected by five centres in the UK and Canada between 1977 and 2005, and almost all ER‐negative and lymph node–positive patients received adjuvant chemotherapy, whereas ER‐positive and/or lymph node–positive patient did not. No patients with HER2 overexpression received trastuzumab.

### 2.2 Immunohistochemistry

Immunohistochemistry was performed on tissue microarrays comprised of single 0.6 mm cores taken from a representative tumour area as assessed on haematoxylin and eosin–stained sections by a specialist breast cancer histopathologist. Immunohistochemical staining was achieved using a Novolink Polymer Detection Kit (Leica) according to the manufacturers’ instructions, the use of which has been described previously. Briefly, tissue was deparaffinized in xylene and rehydrated in ethanol followed by water. Antigen retrieval was performed in 0.01 mol L^−1^ sodium citrate buffer (pH 6.0), with tissue heated for 10 minutes at 750 W in a microwave and then 10 minutes at 450 W. Tissue was blocked with Novolink Peroxidase Block and then Novolink Protein Block solution with Tris‐buffered saline (TBS) washes in‐between. Rabbit polyclonal PKA C‐α antibody (Cell Signalling Technology #4782) was used at a concentration of 1:50 diluted in Bond Primary Antibody Diluent (Leica). Mouse monoclonal PP‐1 alpha antibody (clone 10C6‐3, Life technologies) was used at concentration of 1:100 diluted in Bond Primary Antibody Diluent (Leica). Both antibodies were incubated on tissue for 1 hour at room temperature, and antibody specificity was confirmed by Western blotting using breast cancer cell lysates prior to use. Following antibody incubation, tissue was washed with TBS before application of Novolink Post Primary solution, further TBS washes and Novolink Polymer solution. Immunohistochemical reactions were developed using 3,3′‐diaminobenzidine as the chromogenic substrate and tissue were counterstained with haematoxylin. Tissue was dehydrated in ethanol and fixed in xylene prior to mounting using DPX. Breast tumour composite sections comprising of grade 1 and 2 early‐stage invasive tumours were used as positive and negative controls, and were included with each staining run.

### 2.3 Scoring and statistical analysis

Slides were scanned at 200× magnification using a Nanozoomer Digital Pathology Scanner (Hamamatsu Photonics). Cytoplasmic staining was assessed using a semi‐quantitative immunohistochemical H‐score, where staining intensity within tumour cells was assessed as none (0), weak (1), medium (2) or strong (3) over the percentage area of each staining intensity. Nuclear staining was assessed as the percentage of tumour cells that demonstrated any staining intensity. Greater than 30% of cores for each TMA were double assessed, with both assessors blinded to clinical outcome and each other's scores.

Statistical analysis was performed using IBM SPSS Statistics (version 24). X‐tile was used to determine cut‐points for assessment using breast cancer–specific survival.^23^ Single measure intraclass correlation coefficients were used to determine concordance between scorers with levels above 0.7 that indicated good concordance. The Pearson chi‐squared test of association was used to determine the relationship between categorized protein expression and clinicopathological variables. Survival curves were plotted according to the Kaplan‐Meier method with significance determined using the log‐rank test. Multivariate survival analysis was computed using the Cox regression analysis. All differences were deemed statistically significant at the level of *P* ≤ .05. This study is reported according to REMARK criteria.^24^ Broad Institute Morpheus software was used to visualize data (https://software.broadinstitute.org/morpheus). Expression of PKA and PP1 was compared with DARPP‐32 and Cdk5 which has been described previously.^15^, ^16^

## 3 RESULTS

### 3.1 PKA and PP1 protein staining and frequency and correlation of expression

**FIGURE 1:**
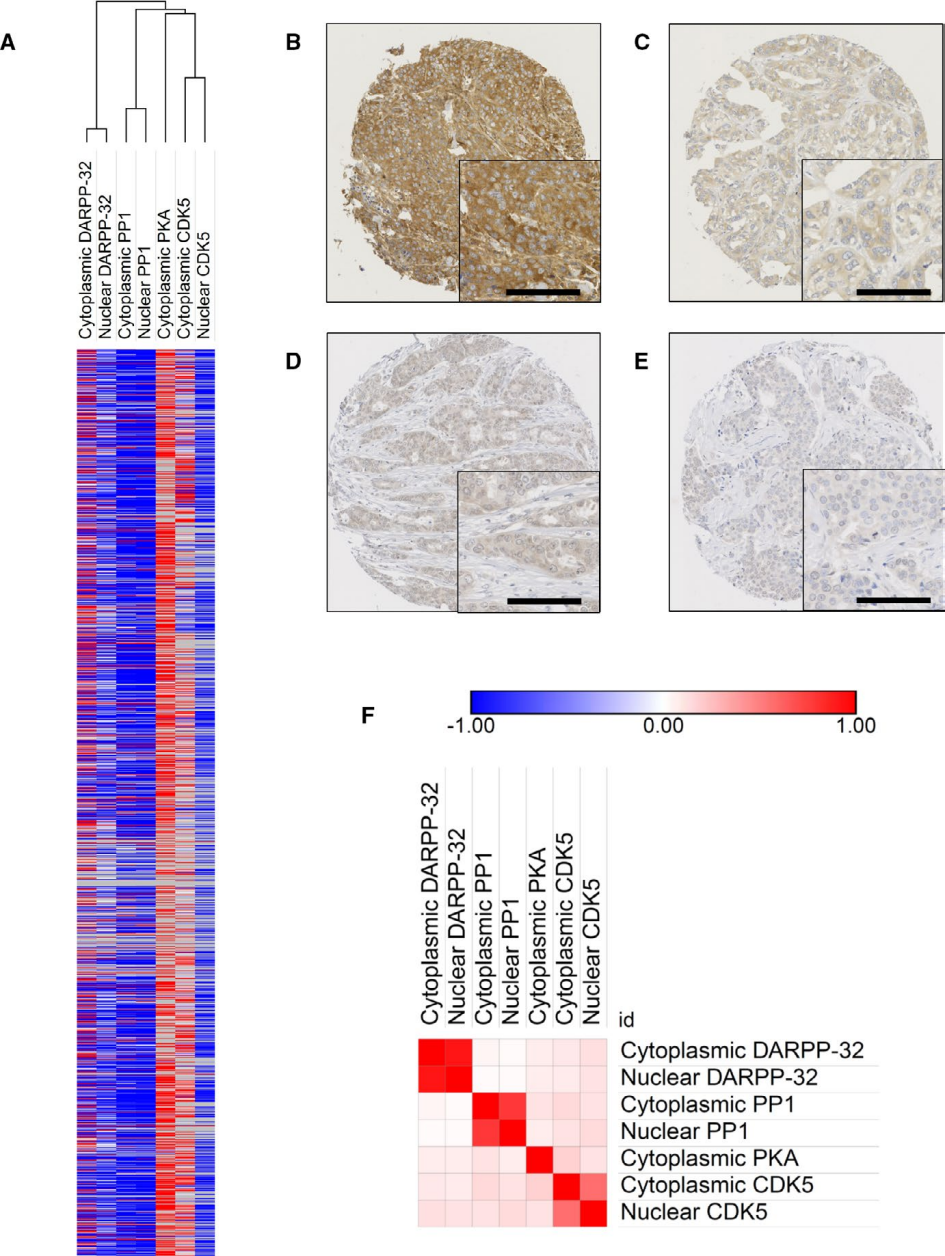
Heat map of DARPP‐32, PP1, PKA and Cdk5 expression (A). Representative photomicrographs of high (B) and low (C) PKA expression, and high (D) and low (E) PP1 expression are shown at 10× magnification with a 20× magnification inset box. Scale bar represents 100 µM. Similarity matrix of Pearson's correlation coefficients between protein expression (F)

The majority of PKA expression was cytoplasmic with weak to strong staining intensity observed in tumour cells; representative staining is shown in Figure 1B,C. The median PKA H‐score was 160 and ranged from 0 to 290; X‐tile computed a H‐score cut‐point of 90, with 89.0% (1032/1159) cases demonstrating high expression. PP1 expression was observed in both the cytoplasm and the nucleus; representative staining is shown in Figure 1D,E. Cytoplasmic PP1 expression had a median H‐score of 0 and ranged from 0 to 160. X‐tile computed a H‐score cut‐point of 25, with 12.6% (187/1488) cases demonstrating high expression. Nuclear PP1 expression had a median H‐score of 0 and ranged from 0 to 100. X‐tile computed a H‐score cut‐point of 20, with 11.9% (178/1491) cases demonstrating high expression. Representative PKA and PP1 protein expression is demonstrated in Figure 1A.

Correlations between the expression of PKA and PP1 together with DARPP‐32 and Cdk5 were assessed. Many of the combinations were statistically significant; however, they were of weak biological significance (Figure 1E). PKA expression was significantly correlated with cytoplasmic (*P* = .012, *R* ^2^ = .077) and nuclear (*P* = .047, *R* ^2^ = .061) PP1 expression. PKA expression was significantly correlated with cytoplasmic (*P* = .030, *R* ^2^ = .066) and nuclear (*P* = .014, *R* ^2^ = .075) DARPP‐32 expression. PKA expression was significantly correlated with cytoplasmic (*P* < .001, *R* ^2^ = .169) and nuclear (*P* < .001, *R* ^2^ = .128) Cdk5 expression. Cytoplasmic PP1 expression was not significantly correlated with cytoplasmic (*P* = .410, *R* ^2^ = .021) and nuclear (*P* = .991, *R* ^2^ = .000) DARPP‐32 expression, but was significantly correlated with cytoplasmic (*P* < .001, *R* ^2^ = .154) and nuclear (*P* = .025, *R* ^2^ = .070) Cdk5 expression.

### 3.2 Relationship between PKA and PP1 protein expression and clinicopathological criteria

Low expression of PKA was significantly associated with the presence of lymphovascular invasion (LVI) (*χ* ^2^ = 6.353, *df* = 1, *P* = .012). PKA expression was not associated with any other clinicopathological variables (Table 1).

**TABLE 1:** Associations between the cytoplasmic and nuclear expression of DARPP‐32, determined in the discovery cohort and validation cohort using immunohistochemistry, with clinicopathological variables

	PKA			PP‐1 cytoplasmic			PP‐1 nuclear		
	Low	High	*P* value	Low	High	*P* value	Low	High	*P* value
Patient age									
<50 y	41 (3.6%)	328 (28.6%)	.919	428 (25.9%)	71 (4.3%)	.140	428 (25.9%)	71 (4.3%)	**.043**
≥50 y	84 (7.3%)	695 (60.4%)		1019 (61.7%)	134 (8.1%)		1032 (62.4%)	124 (7.5%)	
Tumour size									
<2CM	71 (6.2%)	617 (53.7%)	.499	891 (54.0%)	135 (8.2%)	.242	902 (54.5%)	127 (7.7%)	.371
≥2CM	54 (4.7%)	406 (35.4%)		555 (33.6%)	70 (4.2%)		557 (33.7%)	68 (4.1%)	
Tumour grade									
1	21 (1.8%)	144 (12.5%)	.501	231 (14.0%)	30 (1.8%)	.245	229 (13.8%)	32 (1.9%)	.838
2	50 (4.4%)	384 (33.4%)		599 (36.3%)	75 (4.5%)		601 (36.3%)	76 (4.6%)	
3	54 (4.7%)	495 (43.1%)		616 (37.3%)	100 (6.1%)		629 (38.0%)	87 (5.3%)	
Tubule formation									
1	12 (1.1%)	69 (6.1%)	.257	104 (6.3%)	15 (0.9%)	.473	103 (6.3%)	16 (14.1%)	.855
2	29 (2.6%)	297 (26.1%)		418 (25.5%)	67 (4.1%)		429 (26.1%)	58 (3.5%)	
3	82 (7.2%)	648 (57.0%)		914 (55.8%)	120 (87.7%)		914 (55.7%)	121 (7.4%)	
Pleomorphism									
1	2 (0.2%)	10 (0.9%)	.084	21 (1.3%)	0 (0.0%)	**.031**	20 (1.2%)	1 (0.1%)	.594
2	43 (3.8%)	265 (23.3%)		447 (435.7%)	50 (3.1%)		439 (26.8%)	59 (3.6%)	
3	78 (6.9%)	739 (65.0%)		968 (59.1%)	152 (9.3%)		987 (60.1%)	135 (8.2%)	
Mitosis									
1	63 (5.5%)	454 (40.0%)	.233	720 (44.0%)	99 (6.1%)	**.012**	719 (43.9%)	103 (6.3%)	.050
2	18 (1.6%)	208 (18.3%)		280 (17.1%)	25 (1.5%)		281 (17.1%)	24 (1.5%)	
3	42 (3.7%)	351 (30.9%)		434 (26.5%)	78 (4.8%)		444 (27.1%)	68 (4.1%)	
Vascular invasion									
Definite	50 (4.4%)	297 (25.9%)	**.012**	415 (25.2%)	64 (3.9%)	.461	426 (25.8%)	53 (3.2%)	.614
No/probable	75 (6.5%)	726 (63.2%)		1030 (62.4)	141 (8.5%)		1033 (62.5%)	141 (8.5%)	
Stage									
1	74 (6.5%)	649 (56.7%)	.278	926 (56.2%)	118 (7.2%)	.050	931 (56.4%)	113 (6.8%)	.082
2	32 (2.8%)	271 (23.7%)		376 (22.8%)	70 (4.2%)		383 (23.3%)	66 (4.0%)	
3	18 (1.6%)	101 (8.8%)		141 (8.6%)	17 (1.0%)		142 (8.6%)	16 (1.0%)	
Nottingham Prognostic Index									
Good	37 (3.2%)	328 (28.7%)	.303	515 (31.3%)	63 (3.8%)	.315	516 (31.3%)	62 (3.8%)	.597
Medium	60 (5.2%)	526 (46.0%)		704 (42.7%)	111 (6.7%)		716 (43.4%)	102 (6.2%)	
Poor	27 (2.4%)	166 (14.5%)		223 (13.5%)	31 (1.9%)		223 (13.5%)	31 (1.9%)	
ER status									
Negative	21 (1.8%)	215 (18.7%)	.273	274 (16.6%)	43 (2.6%)	.507	281 (17.0%)	36 (2.2%)	.797
Positive	104 (9.1%)	809 (70.4%)		1174 (71.0%)	162 (9.8%)		1180 (71.3%)	159 (9.6%)	
PR status									
Negative	52 (4.8%)	414 (38.3%)	.831	579 (37.1%)	82 (5.3%)	.865	586 (37.5%)	75 (4.8%)	.597
Positive	66 (6.1%)	548 (50.7%)		784 (50.3%)	114 (7.3%)		790 (50.6%)	110 (7.0%)	
HER2 status									
Negative	105 (9.8%)	867 (80.8%)	.978	1249 (80.5%)	176 (11.3%)	.529	1262 (81.2%)	166 (10.7%)	.251
Positive	11 (1.0%)	90 (8.4%)		108 (7.0%)	15.8 (1.2%)		107 (6.9%)	19 (1.3%)	

Note

The *P* values are resultant from Pearson's chi‐squared test of association, and significant values (*P* < .05) are highlighted in bold.

Low cytoplasmic PP1 expression was associated with pleomorphism (*χ* ^2^ = 6.918, *df* = 2, *P* = .031) and mitosis (*χ* ^2^ = 8.849, *df* = 2, *P* = .012). Low nuclear PP1 expression was associated with older patient age (*χ* ^2^ = 4.112, *df* = 1, *P* = .043). Nuclear and cytoplasmic PP1 expression was not associated with any other clinicopathological variables (Table 1).

### 3.3 Association between PKA and PP1 protein expression and survival

**FIGURE 2:**
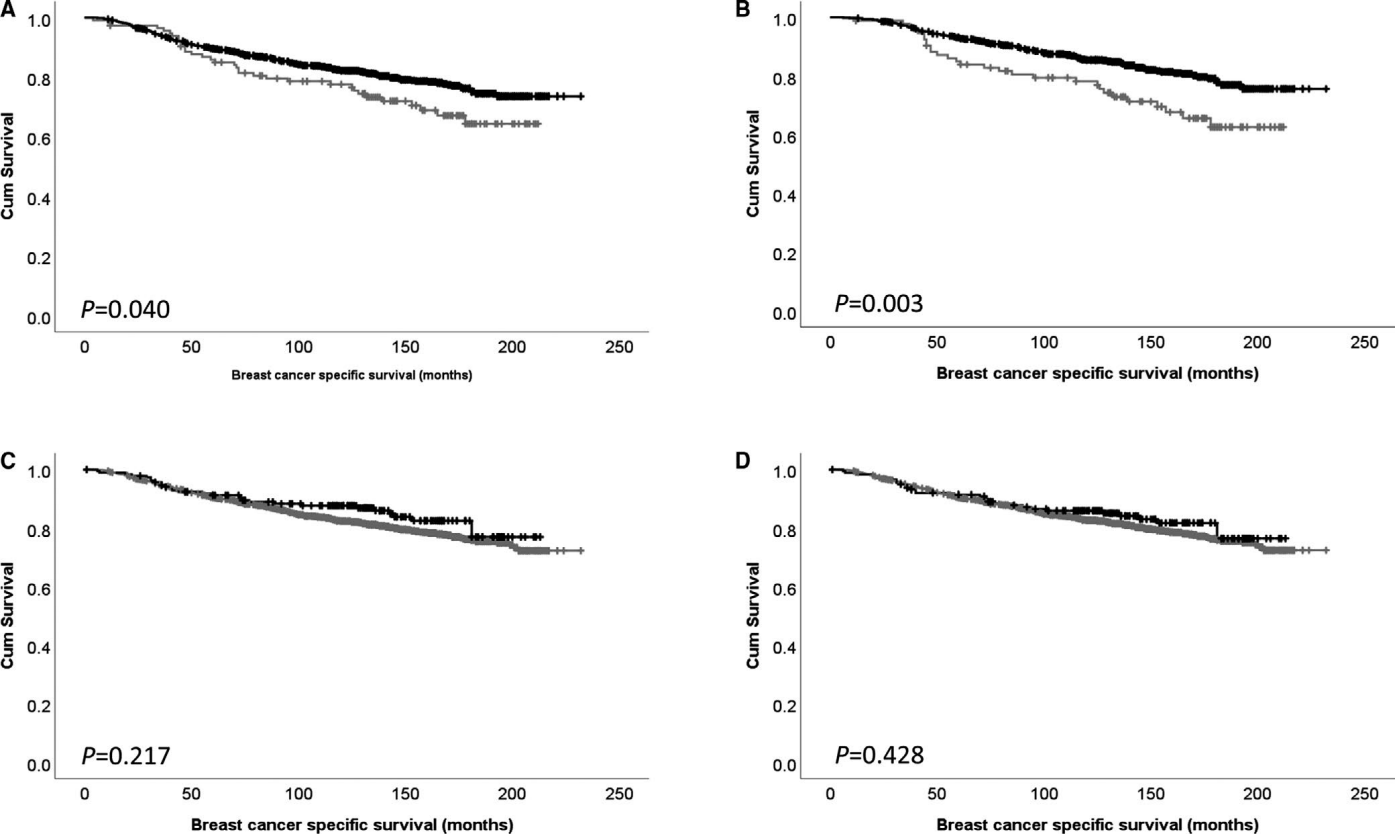
Kaplan‐Meier analysis of PKA (A) and PKA expression in oestrogen receptor–positive breast cancer (B) and cytoplasmic (C) and nuclear (D) PP1 expression where the impact of low (grey line) and high (black line) expression is shown

Low PKA expression was significantly associated with adverse survival of breast cancer patients (*P* = .040) (Figure 2A). Ten‐year survival of breast cancer patients with high expression of PKA was 82.3% (95% confidence interval (CI) = 0.768‐0.848) and low expression of PKA was 77.6% (95% CI = 0.675‐0.835). Expression of PKA did not remain associated with survival when potentially confounding factors of tumour size, grade, nodal stage, ER status, PR status, HER2 status, and LVI status were included in multivariate Cox regression analysis (*P* = .201; hazard ratio (HR)=0.768; 95% CI = 0.513‐1.151) (all with individual log‐rank statistics of *P* < .001). Interestingly, a stronger association was observed between low levels of PKA expression and adverse survival in ER‐positive breast cancer patients (*P* = .003) (Figure 2B).

Cytoplasmic and nuclear PP‐1 protein expression was not associated with survival of breast cancer patients (*P* = .217 and *P* = .428) (Figure 2C,D).

**FIGURE 3:**
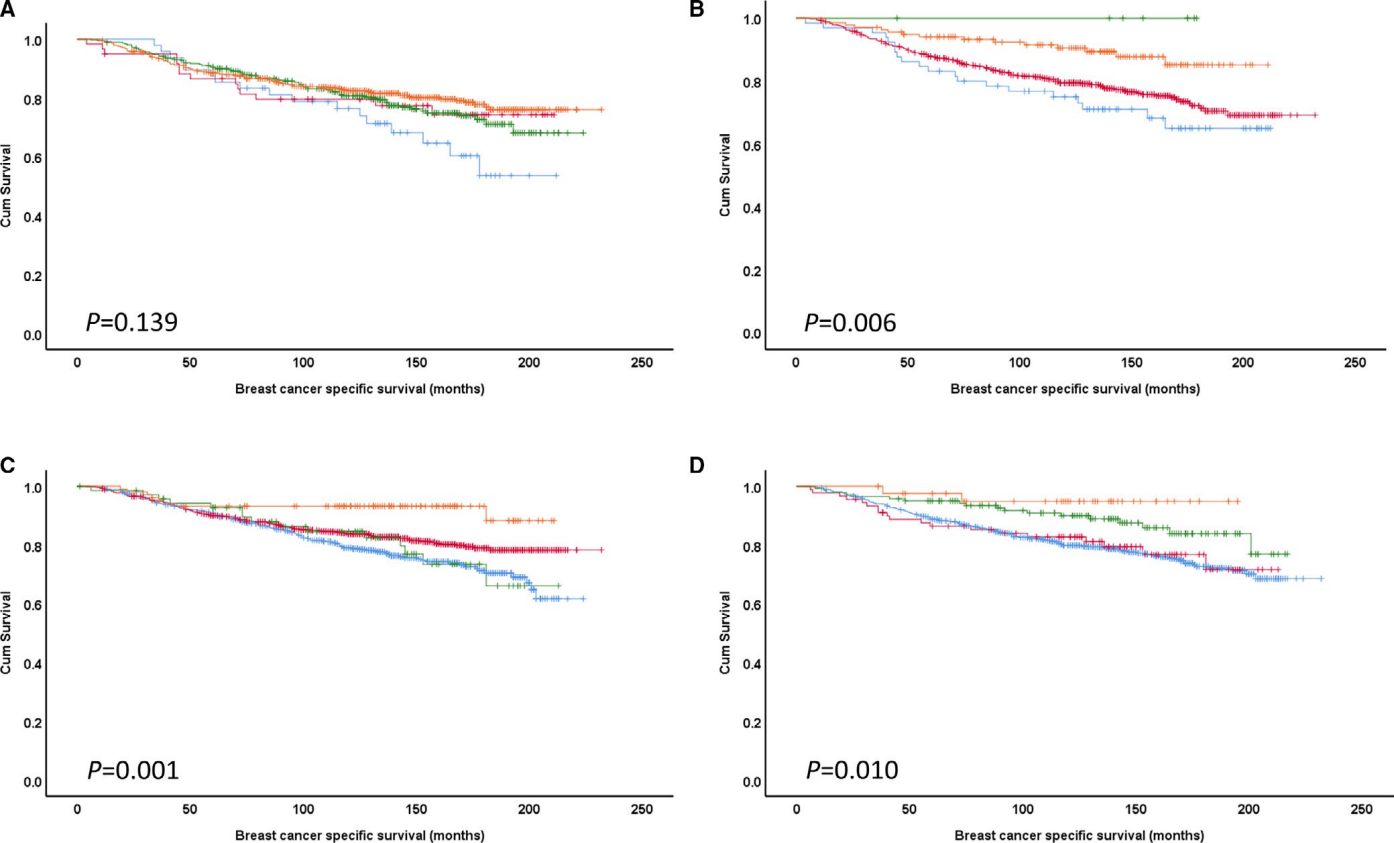
Kaplan‐Meier analysis of combined protein expression. A, PKA and cytoplasmic DARPP‐32 expression; high PKA and high DARPP‐32 (orange), high PKA and low DARPP‐32 (green), low PKA and high DARPP‐32 (pink) and low PKA and low DARPP‐32 (blue) are shown. B, PKA and cytoplasmic Cdk5 expression; high PKA and high Cdk5 (orange), high PKA and low Cdk5 (pink), low PKA and high Cdk5 (green) and low PKA and low Cdk5 (blue) are shown. C, PP1 and cytoplasmic DARPP‐32 expression; high PP1 and high DARPP‐32 (orange), high PP1 and low DARPP‐32 (green), low PP1 and high DARPP‐32 (pink) and low PP1 and low DARPP‐32 (blue) are shown. D, PP1 and Cdk5 expression; high PP1 and high Cdk5 (orange), high PP1 and low Cdk5 (pink), low PP1 and high Cdk5 (green) and low PP1 and low Cdk5 (blue) are shown

Associations between combined PKA, PP1, DARPP‐32 and Cdk5 expression and breast cancer–specific survival were assessed. The combination of low PKA and low Cdk5 expression (*P* = .006; Figure 3B), low PP1 and low DARPP‐32 (*P* = .001; Figure 3C) and combination of low PP1 and low Cdk5 (*P* = .010; Figure 3D) showed significant association with shorter survival. However, the combined expression of PKA and DARPP‐32 was not associated with breast cancer‐specific survival (*P* = .139) (Figure 3A).

### 3.4 PKA and PP1 mRNA expression in the METABRIC cohort

**FIGURE 4:**
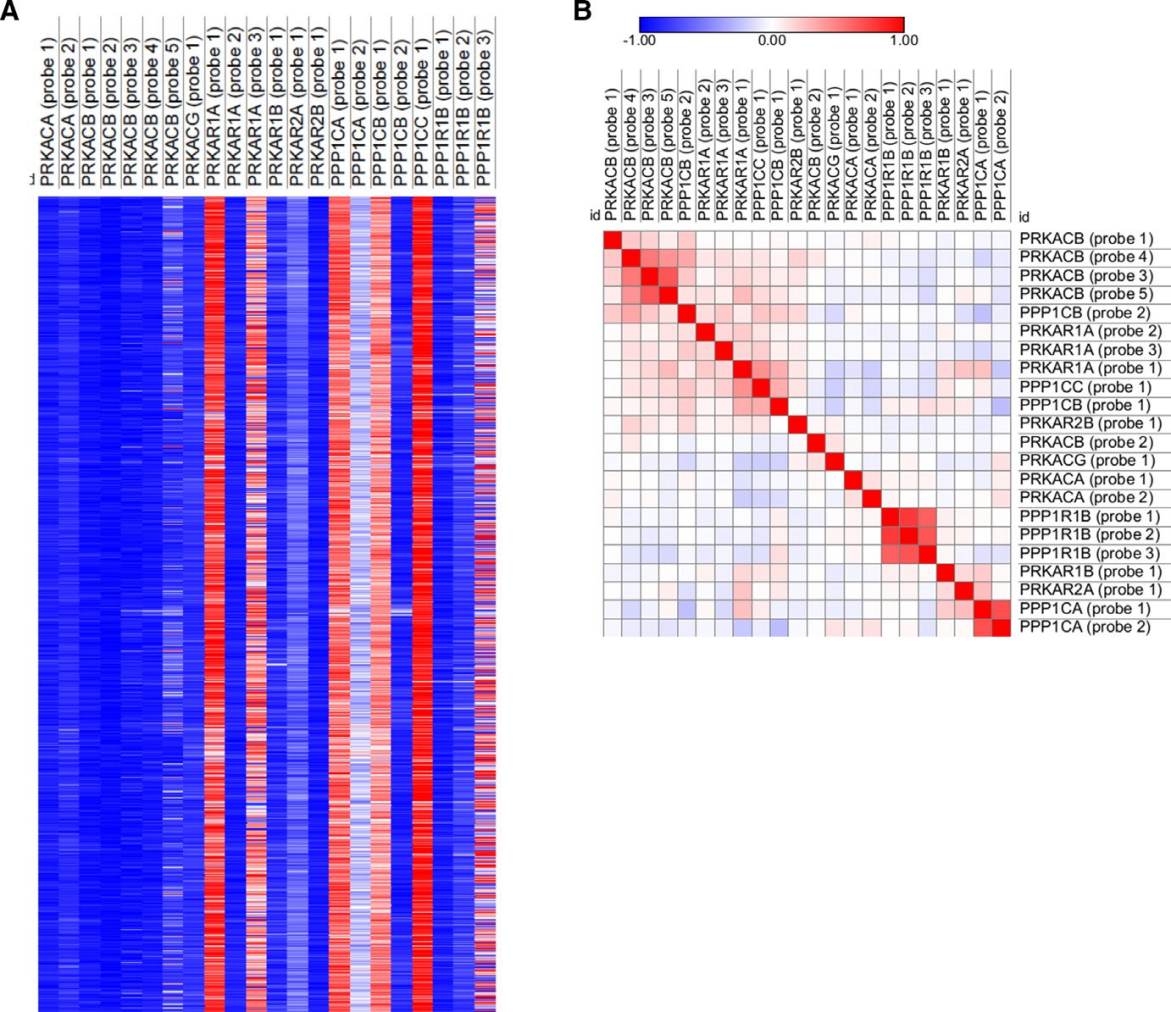
Heat map of *PRKACA*, *PRKACB*, *PRKACG*, *PRKAR1A*, *PRKAR1B*, *PRAKAR2A*, *PRKAR2B*, *PPP1CA*, *PPP1CB*, *PPP1CC*, *PPP1R1B* and *CDK5* expression (A), and Pearson's correlation coefficients between them (B)

PKA and PP1 mRNA expression was assessed for association with patient survival in the METABRIC cohort using probe‐level information. Probe‐level information was used so that correlations with PPP1R1B variants could be assessed. *PPP1R1B* probe 1 (ILMN_1690096) is located within a coding area that corresponds to the N‐terminal region of DARPP‐32; *PPP1R1B* probe 2 (ILMN_1759012) and *PPP1R1B* probe 3 (ILMN_2304495) were both located in untranslated regions (5′ and 3′, respectively). DARPP‐32 mRNA would align with probe 1 and probe 3; t‐DARPP mRNA would align with probe 2 and probe 3. *PRKACA*, *PRKACB*, *PRKACG*, *PRKAR1A*, *PRKAR1B*, *PRKAR2A* and *PRKAR2B* were assessed for PKA and *PPP1CA*, *PPP1CB* and *PPP1CC* were assessed for PP1; heat map visualization of expression and correlation between expression is shown in Figure 4A,B.

Table 2 shows associations between probe‐level information and patient survival in the METABRIC cohort using the Cox (proportional hazards) regression analysis. PRKACG, PRKAR1B and PPP1CA were associated with patient survival. One of five probes for PRKACB, one of three probes for PRKAR1A and one of two probes for PPP1CB were associated with patient survival.

**TABLE 2:** Each gene had probe‐level information assessed against survival using Cox (proportional hazards) regression analysis

Gene	Probe	*P* value	Hazard ratio	95% confidence interval
*PRKACA*	ILMN_1786195	.685	0.892	0.513‐1.552
	ILMN_2395314	.171	1.411	0.862‐2.311
*PRKACB*	ILMN_1690314	**.021**	**0.451**	**0.230‐0.885**
	ILMN_1759922	.189	1.576	0.800‐3.103
	ILMN_1771523	.434	0.874	0.624‐1.224
	ILMN_2278112	.546	0.871	0.556‐1.364
	ILMN_2374234	.161	0.924	0.827‐1.032
*PRKACG*	ILMN_1808368	**.045**	**1.616**	**1.011‐2.582**
*PRKAR1A*	ILMN_1738632	.065	0.898	0.801‐1.007
	ILMN_2277077	**.003**	**0.395**	**0.215‐0.724**
	ILMN_2389590	.146	0.953	0.893‐1.017
*PRKAR1B*	ILMN_1674390	**.001**	**1.612**	**1.205‐2.156**
*PRKAR2A*	ILMN_1681888	.347	0.863	0.635‐1.173
*PRKAR2B*	ILMN_1684850	.465	0.797	0.433‐1.467
*PPP1CA*	ILMN_1695827	**.017**	**1.215**	**1.035‐1.426**
	ILMN_2377980	**>.001**	**1.732**	**1.416‐2.118**
*PPP1CB*	ILMN_2405018	**.003**	**0.772**	**0.651‐0.916**
	ILMN_2405023	.208	0.779	0.527‐1.150
*PPP1CC*	ILMN_1701855	.605	0.977	0.758‐1.175

Note

*P* values, hazard ratio and 95% confidence interval around the hazard ratio are shown. Significant results are highlighted in bold.

## 4 DISCUSSION

This study investigated the expression and co‐expression of four important signalling proteins in breast cancer and assessed their prognostic and clinical significance. We provide evidence that low expression of PKA is significantly associated with adverse survival of breast cancer patients in a large cohort of breast cancer patients; interestingly, this relationship was stronger in ER‐positive breast cancer patients. The ER relationship is interesting, as we have made similar observations with DARPP‐32 expression as part of previous studies.^15^ PP1 expression was not associated with patient survival. Previous studies have linked DARPP‐32 and HER2‐positive breast cancer; our current findings provide further evidence that the DARPP‐32 signalling nexus plays a role in breast cancer subgroups, in particular, in ER‐positive disease.

The function of both PKA and PP1 can be inhibited by DARPP‐32 dependent upon its phosphorylation status. DARPP‐32 can be phosphorylated by PKA on threonine 34 to allow PP1 inhibition or by Cdk5 on threonine 75 to allow inhibition of PKA. Our findings demonstrate that PKA expression was significantly correlated with DARPP‐32 and Cdk5 expression (both determined in previous studies^15^, ^16^). Our findings also demonstrate that PP1 expression was significantly correlated with previously determined Cdk5 expression, but not DARPP‐32 expression. It is important to note that this study assessed the expression level of PKA and PP1 protein and mRNA and inference on cellular activity cannot be made. PP1 expression was assessed in both the cytoplasm and the nucleus, whereas PKA was only observed in the cytoplasm. Neither PP1 nuclear or cytoplasmic expression was associated with patient survival; however, different associations were observed with clinicopathological variables. Cytoplasmic PP1 was associated with pleomorphism and mitosis, indicating an association with altered cell growth, whereas nuclear PP1 was associated with patient age, studies have indicated differential subcellular localization.^25^

In addition to protein expression, mRNA expression of PKA and PP1 subunits was assessed, including *PRKACA*, *PRKACB*, *PRKACG*, *PRKAR1A*, *PRKAR1B*, *PRKAR2A* and *PRKAR2B*, for PKA and *PPP1CA*, *PPP1CB* and *PPP1CC* for PP1. Probe‐level information was assessed so that correlations could be made with represented *PPP1R1B* variants in the data set. Expression of the three PPP1R1B probes were closely related; however, no strong biologically relevant association was observed between expression of the PPP1R1B probes and other probes assessed in this study.

*PRKACG*, *PRKAR1B* and *PPP1CA* were the most strongly and reproducibly associated with breast cancer–specific survival. There were other significant associations between specific probes and survival; one of five probes of *PRKACB*, one of three probes for *PRKAR1A* and one of two probes for *PPP1CB* *PRKACB*, *PRKAR1A* and *PPP1CB* have multiple splice variants, and the observation that not all probes are associated with survival, may indicate that certain transcripts are more important in breast cancer and warrants further investigation.

In breast cancer, low *PRKAR1A* expression in combination with high *SRC* expression has been shown to be associated with basal‐like and HER2‐positive breast cancer associated with poor survival,^3^ and in HER2‐resistant breast cancer, down‐regulation of *PRKAR2A* and *PRKAR2B* has been observed, in combination with an observed increase with *PPP1R1B*.^4^ Our findings suggest that *PRKAR1B* and *PRKACG* were associated with disease‐specific survival of the total patient cohort.

Our findings suggest that *PPP1CA*, *PRKACG* and *PRKAR1B* are associated with breast cancer–specific survival. Previously published studies have indicated a role for PPP1CA in cancer^26^, ^27^; in breast cancer, PPP1CA may play a role in cancer stem cell populations.^28^ Although a role for *PRKACG* and *PRKAR1B* in breast cancer has not been described, PRKACG has been shown to act as a RUNX1‐mutation associated hub gene in acute myeloid leukaemia^29^ and single nucleotide polymorphisms of PRKAR1B are associated with inferior survival of advanced renal cell cancer patients.^30^

In previous studies, we demonstrated that low DARPP‐32 and Cdk5 expression is associated with adverse survival of breast cancer patients.^15^, ^16^ We hypothesized that a reduction in DARPP‐32 and an increase in t‐DARPP in breast cancer result in a concomitant alteration of PKA and PP1 signalling. In this study, we explored associations between PKA, PP1, DARPP‐32 and Cdk5 expression and breast cancer–specific survival. The combination of PKA and Cdk5 expression was significantly associated with survival as was the combined expression of PP1 and DARPP‐32 and the combined expression of PP1 and Cdk5. In patients with high expression of DARPP‐32, low expression of PP1 was associated with adverse survival when compared to high expression in the same group. In patients with high expression of Cdk5, high expression of PKA was associated with adverse survival when compared with low expression in the same subgroup; low expression of PP1 was significantly associated with adverse survival when compared with high expression in the same subgroup. These findings warrant further functional investigation of the DARPP‐32 signalling nexus in breast cancer, with a particular focus in ER‐positive disease.

## 5 CONCLUSION

Low PKA expression was significantly associated with adverse survival of breast cancer patients in a large cohort of breast cancer patients; interestingly, this relationship was stronger in ER‐positive breast cancer patients. PP1 protein expression was not associated with patient survival. PKA and PP1 subunit mRNA expression was also assessed; *PPP1CA*, *PRKACG* and *PRKAR1B* were associated with breast cancer–specific survival. PKA expression and PP1 expression are of significant interest in cancer as they are involved in a wide array of cellular processes and may play an important role in patient outcome.

## CONFLICT OF INTEREST

The authors declare no conflict of interest.

## AUTHOR CONTRIBUTION

**Behnaz Saidy:** Data curation (equal); formal analysis (equal); investigation (equal); writing – review and editing (equal). **Shreeya Kotecha:** Data curation (equal); investigation (equal); writing – review and editing (equal). **Anna Butler:** Data curation (equal); investigation (equal); writing – review and editing (equal). **Emad A. Rakha:** Resources (equal); writing – review and editing (equal). **Ian O. Ellis:** Resources (equal); writing – review and editing (equal). **Andrew R. Green:** Resources (equal); writing – review and editing (equal). **Stewart G. Martin:** Project administration (equal); resources (equal); supervision (equal); writing – review and editing (equal). **Sarah J. Storr:** Conceptualization (lead); data curation (equal); formal analysis (lead); funding acquisition (lead); investigation (equal); methodology (equal); project administration (equal); resources (equal); supervision (equal); validation (equal); visualization (equal); writing – original draft (lead); writing – review and editing (equal).

## ETHICAL APPROVAL

Ethical approval was granted by Nottingham Research Ethics Committee 2, under the title ‘Development of a molecular genetic classification of breast cancer’ (C202313). METABRIC samples were collected by five centres in the UK and Canada and were acquired with appropriate consent from the respective institutional review boards. All procedures performed in studies involving human participants were in accordance with the ethical standards of the institutional and/or national research committee and with the 1964 Helsinki Declaration and its later amendments or comparable ethical standards. All samples collected from Nottingham used in this study were pseudo‐anonymized; those collected prior to 2006 did not require informed patient consent under the Human Tissue Act; after 2006, informed consent was obtained from all individual participants included in the study.

## Data Availability

The METABRIC data set is publically available at https://www.ebi.ac.uk/ega/studies/EGAS00000000098. Immunohistochemistry data sets analysed during the current study are available from the corresponding author on reasonable request.
